# Sunset Yellow Dye Induces Amorphous Aggregation in β-Lactoglobulin at Acidic pH: A Multi-Techniques Approach

**DOI:** 10.3390/polym14030395

**Published:** 2022-01-20

**Authors:** Javed Masood Khan, Ajamaluddin Malik, Fohad Mabood Husain, Mohammed J. Hakeem, Abdullah S. Alhomida

**Affiliations:** 1 Department of Food Science and Nutrition, College of Food and Agricultural Sciences, King Saud University, Riyadh 11451, Saudi Arabia; fhusain@ksu.edu.sa (F.M.H.); mhakeem@ksu.edu.sa (M.J.H.); 2 Department of Biochemistry, College of Science, King Saud University, Riyadh 11451, Saudi Arabia; amalik@ksu.edu.sa (A.M.); alhomida@ksu.edu.sa (A.S.A.)

**Keywords:** β-lactoglobulin, sunset yellow, amorphous aggregation, acidic pH, whey protein

## Abstract

Protein aggregation is of two types: (i) amorphous and (ii) amyloid fibril. Several extrinsic factors (temperature, pH, and small ligands) stimulate protein aggregation in vitro. In this study, we have examined the role of sunset yellow (SY) on the β-lactoglobulin (BLG) aggregation at pH 2.0. We have used spectroscopic (turbidity, Rayleigh light scattering (RLS), far-UV CD) and microscopic (transmission electron microscopy [TEM]) techniques to describe the effects of SY on BLG aggregation. Our results showed that BLG aggregation is dependent on SY concentrations. Very low concentrations (0.0–0.07 mM) of SY were unable to induce aggregation, while SY in the concentrations range of 0.1–5.0 mM induces aggregation in BLG. The kinetics of SY-stimulated aggregation is very fast and monomeric form of BLG directly converted into polymeric aggregates. The kinetics results also showed SY-induced BLG aggregation disappeared in the presence of NaCl. The far-UV CD and TEM results indicated the amorphous nature of SY-induced BLG aggregates. We believe that our results clearly suggest that SY dye effectively stimulates BLG aggregation.

## 1. Introduction

Amorphous protein aggregates do not form any specific higher-order structure; is nevertheless believed to be related to diseases. Few reports suggest that the amorphous aggregate is causing diseases also [1,2]. For example, amorphous protein aggregates that coat neuro-fibrillar tangles are found in Alzheimer’s patients [3]. In Parkinson’s, the α-synuclein is aggregated and forms both amyloid fibril and amorphous aggregates [4]. The P23T γD-crystallin protein forms amorphous aggregates and causes cataracts [5]. Renal failure was also occurring due to the formation of amorphous aggregates of immunoglobulin protein [6]. Several other proteins that are not related to diseases also form amyloid fibril or amorphous aggregates under in vitro conditions [7,8,9]. Several stimulating factors such as temperature, pH, ionic strength, string, surfactant, lipid, and food dyes were reported to stimulate native soluble protein to form well-defined amyloid or amorphous aggregates [10,11]. The mechanism of food dye-induced aggregation was not studied in detail, but a few research articles were available regarding its mechanism of aggregation induction [12]. Numerous food dyes have been tested for their ability to induce amyloid or amorphous aggregation in proteins [13,14]. Tartrazine induces the amyloid fibril in human serum albumins (HSA) and bovine serum albumin (BSA) at pH 3.5, and bigger size aggregates were formed in BSA [15]. Apart from aggregation-inducing property, many research articles are published in which it was reported that food dyes, particularly Amaranth and Tartrazine, are inhibit the amyloid fibrillation of hen egg white lysozyme [16]. Carmoisine is an azo dye that binds with hen egg white lysozyme and shows an inhibitory effect on amyloid fibrillation in lysozyme [17]. Sunset Yellow (SY) and Ponceau 4R have also interacted with lysozyme at acidic pH and inhibiting temperature-induced amyloid fibrillation [18]. Keeping this in mind, we have evaluated the mechanism of interaction and amyloid-inducing property of SY on Beta-lactoglobulin (BLG) protein at acidic pH.

**Figure 1:**
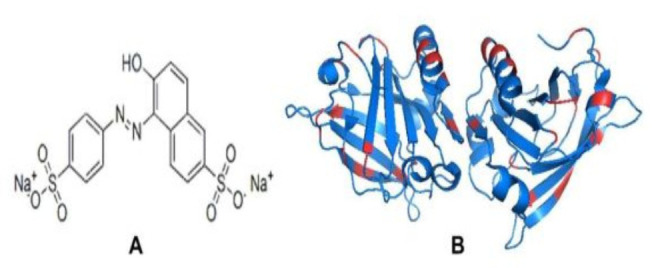
Chemical structure of sunset yellow dye (SY) (**A**) and (**B**) molecular structure of BLG (PDB ID: 2AKQ). The basic residues are highlighted in red.

SY is a dianionic monoazo dye having two sulphate groups (Figure 1A) usually used in the food and cosmetics industries [19]. SY is used in several foods, like confectionery products, sweets, savory samples, ice candy, and beverages, medicines, cosmetics, and dietary supplements, etc. [20]. The dietary exposure of SY in different countries lies in between 0.3–6.7 mg/kg. SY does not cause any adverse effects in mice and rats, as confirmed by acute, chronic studies [21]. Furthermore, SY does not show any mutagenesis or carcinogenic activity in vitro or in vivo [22]. Moreover, some reports suggest that the dietary exposure of SY causes problems in the reproductive and neurobehavioral parameters of mice [23]. It strongly interacted with human serum albumin compared to natural dyes [24]. The interaction of food dyes “SY” with food proteins like Beta-lactoglobulin (BLG) is important because these two molecules are present together in several foods.

BLG (18.4 kDa) is a small globular protein abundantly present in milk’s whey fraction. BLG belongs to the lipocalin family protein and is involved in ligand binding. BLG can bind hydrophobic molecules in vitro, and it is believed that it is transported to the insoluble and/or chemically sensitive ligand from mother to offspring [25]. BLG has a barrel structured by eight antiparallel β-strands and one α-helix, which is present on the outer surface of the barrel (Figure 1B) [26]. BLG is one of the best-known lipid-binding proteins and can interact with many small molecules, such as fatty acids, retinol, etc. [27]. BLG forms amyloid-like aggregates at a temperature higher than 75 °C and low pH [28]. BLG is highly used in the food industry because of its fibrils-forming properties. The BLG fibrils increase viscosity and gel strength, which can be further used in the food industry [29]. In a previous paper, we have shown that the food dye “Allura red” induces SDS-soluble amyloid-like aggregates in BLG protein at acidic pH [14]. Generally, BLG fibrillation needs a very harsh condition to develop. We are trying to establish easy and gentle methods to induce amyloid fibril formation in BLG protein.

Herein, we assessed the effect of SY on BLG aggregation at acidic pH and tried to propose the mechanism of SY action. Until now, BLG aggregation in the presence of the food dye “SY” has not been discussed. We have tried to characterize the nature of BLG aggregates formed in the presence of SY dye. The effect of salt on the BLG aggregation pathway was also elaborated. Turbidity and Rayleigh light scattering, far-UV CD, and transmission electron microscopy (TEM) were used to characterize the nature of SY-induced aggregation. The results of this work will provide a complete understanding of SY-induced BLG aggregation. This work will also provide the method to induce aggregation in BLG protein and utilize the aggregates for food processing. Many proteins, such as milk, legume, egg, and cereal, form amorphous or amyloid fibril at different food processing conditions [30,31].

## 2. Materials and Methods

### 2.1. Materials

Bovine β-Lactoglobulin (BLG) (lot#SLBP8394 V) and Sunset Yellow (SY) dye (Lot#SHBL0658) were purchased from Sigma—Chemicals Co. (St. Louis, MO, USA). All other chemicals used in this study were of analytical grade. The data shown in this article are statistically significant.

#### 2.1.1. Solution Preparation

A 5.0 mg/mL BLG was dissolved in 20 mM phosphate buffer pH 7.4. The concentration of BLG was calculated spectrophotometrically by using an extinction coefficient of 17,600 cm^−1^ M^−1^ at 280 nm [32]. SY (20 mM) dye stock was made in MilliQ (Millipore Corporation, Bedford, MA, USA) water. All solutions used in this study were freshly prepared.

#### 2.1.2. Turbidity and Rayleigh Scattering Measurements

The turbidity and Rayleigh light scattering (RLS) measurements were carried out to detect the SY dye-induced aggregation of BLG at pH 2.0. The turbidity was measured by Cary 60 UV–vis Spectrophotometer (Agilent technologies, Inc. USA, Santa Clara, CA, USA) and Cary Eclipse Fluorescence Spectrofluorometer Agilent technologies, Inc. USA, was used to perform the RLS study. The turbidity of aggregated and non-aggregated samples was measured by taking absorbance at 650 nm. However, the RLS was measured of SY-induced aggregated samples by excitation of samples at 650 nm and the emission of the samples was recorded at 650 nm. BLG (0.2 mg/mL) samples were treated with different SY concentrations (0.0 to 5.0 mM) at pH 2.0. The data of turbidity and RLS was plotted against SY concentrations.

The turbidity was also measured of samples containing BLG (0.2 mg/mL) treated with 0.2 mM SY dye concentrations at different pHs.

#### 2.1.3. SY Dye-Induced Aggregation Kinetics

RLS measured the SY dye-induced BLG aggregation kinetics at pH 2.0 in three different conditions.

In the first set, the RLS was measured of samples in which the BLG concentration (0.2 mg/mL) was fixed, and the effect of varying concentrations of SY dyes (0.01, 0.05, 0.1 and 0.2 mM) was seen.

In the second set, the light scattering of different concentrations of 0.05, 0.1, 0.2, 0.5, and 1.0 mg/mL treated with 0.2 mM of SY dye was measured against time in seconds.

In the third set, the effect of salt was seen in SY-induced BLG aggregation kinetics. In this condition, the BLG (0.2 mg/mL) and SY (0.2 mM) concentrations were fixed, while the effect of different concentrations of salts (0, 100, 150, 200, and 500 mM) on BLG aggregation kinetics was seen at pH 2.0. The RLS kinetics was measured on Cary Eclipse Fluorescence Spectrofluorometer (Inc. USA, Santa Clara, CA, USA) after excited at 650 nm, and emission was taken at 650 nm. The emission was plotted at 650 nm against time in seconds. The excitation and emission slit widths were 1.5 and 2.5 nm, respectively.

#### 2.1.4. Circular Dichroism (CD)

The change in the secondary structure of BLG in response to SY dye was determined using a J-1500 spectropolarimeter (JASCO, Tokyo, Japan). The BLG (0.2 mg/mL) was treated with different concentrations of SY dye (0.0–5.0 mM) at pH 2.0. Before CD measurements, the SY-treated BLG samples were centrifuged at 5000 rpm for 10 min to remove the excess free dye. The centrifugation was done to avoid noise in CD measurements. For far-UV CD measurements, 450 μL SY-treated BLG samples were poured into a 1 mm path-length quartz cuvette, and spectra were measured in the range of 200 to 250 nm, using a scanning speed of 100 nm min^−1^ and a bandwidth of 2 nm, at room temperature. The final spectrum of each sample was averaged based on three accumulations. The signal of the buffer was subtracted manually. In other measurements, the BLG was treated with fixed concentrations (0.2 mM) of SY dye and then different concentrations of NaCl was added to see the solubility of BLG.

#### 2.1.5. Transmission Electron Microscopy (TEM)

The morphology of SY-induced BLG aggregates was tested by TEM imaging. For TEM measurements, 5.0 µL of SY-BLG aggregates solution was transferred to Formvar and carbon-coated nickel grids and incubate for 5.0 min. The aggregate pour grids were then washed four to five times with MilliQ water and then negatively stained with 2% *w/v* uranyl acetate dye and keep the samples were kept in the desiccator for two to three days for drying. Dried grids were viewed using a JEOL transmission electron microscope (JEOL, Tokyo, Japan).

## 3. Results

### 3.1. Turbidity

**Figure 2:**
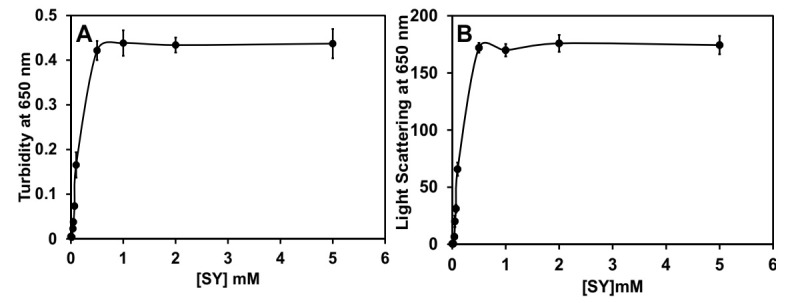
Aggregation profile of BLG against SY dye at pH 2.0. (**A**) Turbidity and (**B**) RLS of BLG vs. different SY dye concentrations in millimolar. The turbidity was captured at 650 nm. The light scattering was recorded at 650 nm after excitations of samples at the same wavelength. BLG concentrations were fixed at 0.2 mg/mL in both measurements.

The effect of SY on the aggregation of BLG at low pH was monitored by turbidity measurements at room temperature. The turbidity was performed by measuring the absorbance of every sample in the absence and presence of different concentrations of SY at 650 nm. It is considered that the absorbance at 650 nm is related to the process of protein aggregation in the solutions. The turbidity profile of BLG treated with SY is summarized in Figure 2A. From the figure, it was evident that the BLG at pH 2.0 without SY does not show any turbidity (Table 1), which confirms that BLG itself does not form any aggregates at this pH. However, the BLG samples treated with different SY concentrations (0.0–5.0 mM) showed significant turbidity at some concentrations of SY. For the BLG incubated with 0.01–0.07 mM SY dye, no turbidity was seen, but in the exposure of concentrations above 0.07 mM, a huge increase in turbidity was recorded, and maximum turbidity was found to be 0.5 mM SY. The threshold of turbidity was found constant above 0.5 mM SY concentration. The turbidity data suggest that BLG starts forming aggregates in the presence of 0.1 mM SY, and maximum aggregation was found in the presence of 0.5 mM SY. Table 1 showed that the control samples (BLG without SY dye at pH 3.5 and SY dye without BLG) do not show any turbidity, which confirms that the turbidity arises when BLG become aggregated.

### 3.2. Rayleigh Light Scattering (R LS) Measurement

RLS was carried out to trace the formation of aggregates in BLG in response to SY dye at acidic pH. RLS is a very sensitive technique to detect the protein aggregation in solution by measuring the light scattering at 650 nm. The SY concentration-dependent changes in the BLG aggregation were monitored by light scattering, and the changes in the light scattering profile are shown in Figure 2B. The sample containing BLG without dye showed no scattering, while in the presence of SY concentrations above 0.07 mM, a rapid increase in scattering was recorded, which confirms that the BLG form aggregates in the presence of SY dye. In the presence of a concentration of ≤0.07 mM, the light scattering was almost zero, which confirms that the BLG was not forming any aggregates in the presence of lower SY concentrations. The SY without BLG and BLG without SY do not show any scattering, confirming that the above controls do not form aggregates as shown in Table 1.

### 3.3. Kinetics of SY-Induced Aggregation

#### Effects of SY on BLG Aggregation

**Figure 3:**
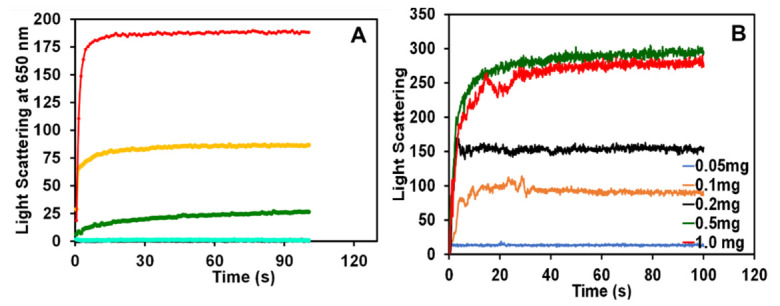
The kinetics of BLG aggregation induced by SY dye were detected by RLS measurements. (**A**) BLG (0.2 mg/mL) treated without (-●-) and with 0.01 (-●-), 0.05 (-●-), 0.1 (-●-), and 0.2 (-●-) mM SY dye at pH 2.0. (**B**) Effects of various concentrations of BLG, 0.05 (▬), 0.1 (▬), 0.2 (▬), 0.5 (▬) and 1.0 mg/mL (▬), on aggregation kinetics with a fixed concentration 0.2 mM of SY dye at pH 2.0. The change in light scattering was measured with respect to time in seconds.

To find out the kinetics of BLG aggregation in the presence and absence of SY dye at pH 2.0, we have measured the light scattering at 650 nm of BLG aggregation in the presence of 0.0, 0.01, 0.05, 0.1, and 0.2 mM of SY concentration shown in Figure 3A. For the BLG (0.2 mg/mL) at pH 2.0 in the absence of SY dye, light scattering was not seen, indicating that BLG itself is not forming aggregates. Light scattering was also not seen in the sample treated with 0.01 mM of SY dye, confirming that low concentrations of SY cannot induce aggregation in BLG protein. However, in the presence of 0.05, 0.1 and 0.2 mM SY dye, an increase in light scattering was seen, but the level of scattering is dependent on the concentrations of SY dye. The SY dye-induced BLG aggregation kinetics are very fast, and no detectable lag phase was found at any SY concentration. The SY-induced aggregation started with a rapid growth phase and shortly reached the saturation phase. The kinetics results suggest that the BLG monomer was directly converted into bigger aggregates without any nucleus formation.

The effect of BLG concentration on the SY-induced aggregation was also explored and data are shown in Figure 3B. The effect of increasing concentrations of BLG in the presence of 0.2 mM SY dye was seen. The kinetics data found that the BLG with concentrations of 0.05 mg/mL in the presence of 0.2 mM SY dye does not show any aggregation. However, in the presence of 0.1, 0.2, 0.5, and 1.0 mg/mL, light scattering was seen and the light scattering profile is dependent on BLG concentration. The aggregation rate was fast at 0.1 to 1.0 mg/mL BLG concentration. The kinetic spectra of 0.5 and 1.0 mg/mL BLG were nearly overlapping, indicating that the aggregation reaction was saturated at 0.5 mg/mL BLG. The only difference was recorded in the extent of aggregation (light scattering intensity) in the kinetics pattern. These kinetics data suggest that the BLG concentrations play an important role in the SY dye-induced aggregation pathways.

**Figure 4:**
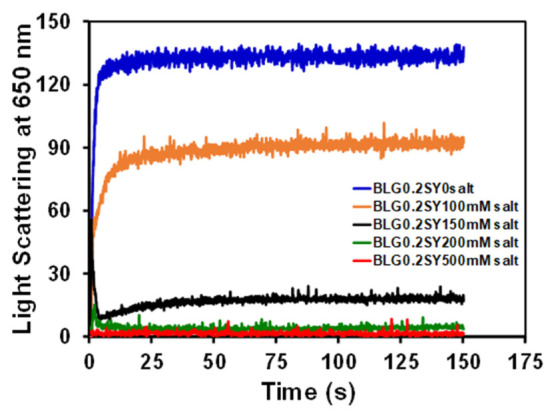
Effects of salts was seen on SY dye-induced BLG aggregation kinetics. BLG (0.2 mg/mL) aggregation kinetics induced by SY in the presence of different concentrations of 0.0 (▬), 100 (**▬**), 150 (**▬**), 200 (**▬**) and 500 (**▬**) mM salts (NaCl) at pH 2.0.

Further, we have seen the role of NaCl on SY-induced BLG aggregation kinetics. The effect of different concentrations of NaCl (0.0–500 mM) was seen on the BLG aggregates (BLG + 0.2 mM SY) shown in Figure 4. From the above kinetics measurements, it was confirmed that the BLG form bigger-sized aggregates in the presence of 0.2 mM of SY concentrations and the aggregation kinetics process was very fast. In the same conditions, the effect of NaCl was evaluated. From Figure 4, it was evident that the aggregates form in the sample BLG with 0.2 mM SY dye alone is similar to Figure 3. Next, the different concentrations of salts were added, and the kinetics were measured. The kinetics results showed that the kinetics pattern of BLG aggregation in the presence of 100 and 150 mM NaCl was not changed, while the light scattering profile started decreasing in response to salt concentrations. In the presence of 100 mM of NaCl, the light scattering was slightly reduced compared to the sample without salts. Moreover, a complete loss of light scattering was seen when aggregated samples were treated with 200 and 500 mM of salts. The kinetics data suggest that the salt is solubilizing the SY-induced BLG aggregates. The possible cause of suppression of BLG aggregation in the presence of salts is interference in electrostatic interaction.

### 3.4. Circular Dichroism Detection

**Figure 5:**
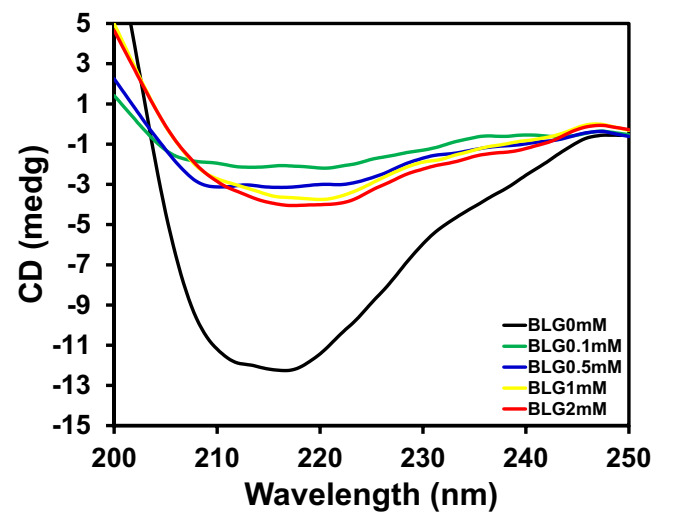
Secondary structure changes were measured by Far-UV CD. BLG (0.2 mg/mL) at pH 2.0 was incubated with different concentrations of SY dye. Far-UV CD spectra of BLG (0.2 mg/mL) in the absence of SY dye (**▬**) and in the presence of 0.1 (▬), 0.5 (▬), 1.0 (▬) and 2.0 (▬) mM SY dye at pH 2.0.

CD spectroscopy is a useful spectroscopic technique usually used to investigate proteins’ secondary structural changes. The protein secondary and tertiary structure can be modified by binding to the small molecules or ligands and sometimes by perturbing the structures of proteins. Far UV-CD measurements were performed with and without the SY and characterized the modification of the secondary structure of BLG. The far-UV spectra of BLG with and without SY were shown in Figure 5. The figure noted that the CD spectrum of native BLG exhibited a broad negative minimum in between 210–220 nm, which is consistent with the other published report, indicating that BLG has a β-sheet structure [33]. When BLG samples were incubated with different concentrations of SY dye (0.1, 0.5, 1.0, and 2.0 mM), a huge decrease in negative ellipticity was recorded without the shift of peak position, which is indicative of a loss of secondary structure. The reduction in negative ellipticity without the peak position shift indicates that the BLG molecules lost the secondary structure in the presence of SY dye. The aggregation caused by SY dye also suggests that the BLG aggregates do not have any ordered secondary structure. It could be a signature of amorphous aggregates. The percent secondary structure changes of BLG in response to SY dye was shown in Table 2.

### 3.5. Morphology of SY-Induced Aggregate by TEM

**Figure 6:**
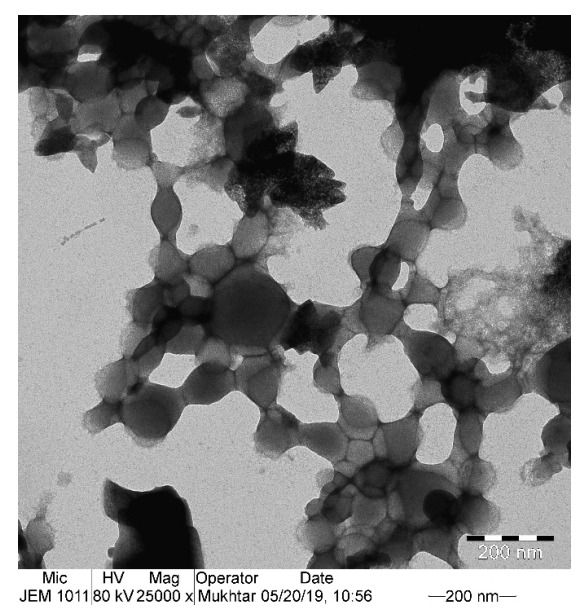
Morphology of BLG aggregates was checked by transmission electron microscopy. Electron micrograph of BLG treated with 1.0 mM SY dye at pH 2.0.

TEM was used to assess the role of SY dye on BLG aggregation. TEM is a powerful technique used to characterize the morphology of protein aggregates and distinguish the fibril and amorphous structure. BLG (0.2 mg/mL) was incubated with and without SY dye overnight at room temperature and acidic pH to form aggregates. The TEM image showed that the BLG without SY dye does not have any visible aggregate structure (image not shown). The BLG treated with 0.5 mM SY dye has shown no specific order structure, which could be an amorphous aggregate structure (Figure 6). The TEM result confirmed that the SY dye interacted with BLG and inducing aggregates and aggregates are amorphous in nature.

### 3.6. Effect of pH on SY-Induced BLG Aggregation

**Figure 7:**
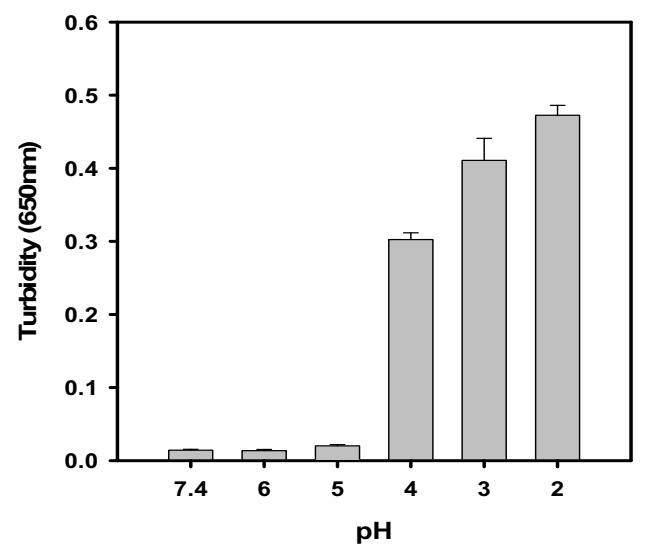
**The** effect of pH was seen on SY-induced BLG aggregation. The turbidity at 650 nm was measured of the BLG (0.2 mg/mL) samples incubated in the presence of 0.2 mM SY at different pHs.

The change in turbidity of SY-induced BLG aggregates was tested and shown in Figure 7. From the figure, it was observed that the turbidity was not seen up to pH 5.0, but below pH 5.0, a progressive increase in turbidity was seen and maximum turbidity was recorded at pH 2.0. The possible cause of the increase in turbidity is due to BLG aggregation. The isoelectric point of BLG is reported at around 5.2, and below pH 5.2, the BLG will be positively charged and have sufficient affinity to interact with the negatively charged sulphate of SY dye.

### 3.7. Effect of NaCl Was Seen on Secondary Structure Modification of SY-Induced BLG Aggregates

**Figure 8:**
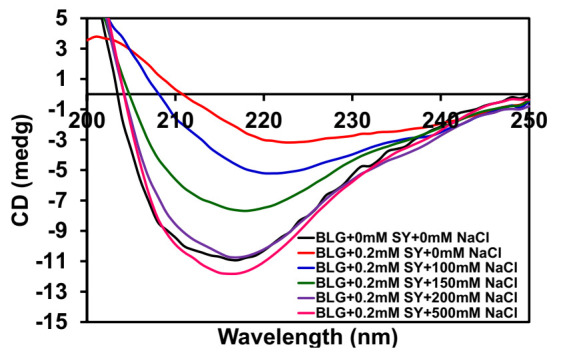
The effect of NaCl was seen on aggregated BLG secondary structure. Far-UV CD spectra of BLG (0.2 mg/mL) was measured at the following conditions: BLG at pH 2.0 (─), BLG + 0.2 mM SY + pH 2.0 + 0.0 mM NaCl (─), BLG + 0.2 mM SY + pH 2.0 + 100 mM NaCl (─), BLG + 0.2 mM SY + pH 2.0 + 150 mM NaCl (─), BLG + 0.2 mM SY + pH 2.0 + 200 mM NaCl (─), and BLG + 0.2 mM SY + pH 2.0 + 500 mM NaCl (─).

The effect of increasing concentration of NaCl was seen on the secondary structure of SY-induced BLG aggregates, which was measured by far-UV circular dichroism, and the data are shown in Figure 8. The BLG at pH 2.0 showed a broad negative minima in the range of 210–220 nm, indicative of β-sheeted protein. As we have already discussed, there was a change in the secondary structure of BLG in the presence of SY dyes. In this measurement, BLG treated with fixed concentrations of (0.2 mM) SY dye and the effects of different concentrations of NaCl were explored. The negative ellipticity of BLG is greatly reduced in the presence of 0.2 mM of SY dye, which indicates the loss of secondary structure. However, in the presence of increasing concentrations of NaCl, the regaining of BLG ellipticity was recorded. The regaining of negative ellipticity is dependent on NaCl concentrations. The regaining of negative ellipticity starts in the presence of 100 mM NaCl concentrations, and nearly native-like structure in BLG was regained at 200 mM NaCl. Further increases in salt concentration (500 mM NaCl) keep the BLG in a native-like state. The far-UV CD data clearly suggest that the BLG was refolded in the presence of 200 mM NaCl.

## 4. Discussion

This study presents experimental evidence of the BLG aggregation stimulated by SY dye at acidic pH. It was extensively reported that anionic molecules like surfactant, lipid, food dyes, and glycosaminoglycan are stimulated protein aggregation [14,34,35]. Generally, these negatively charged molecules interact electrostatically with proteins and modulate aggregation [36]. This study found that the anionic food dye “SY” triggered BLG aggregation at acidic pH. Several spectroscopic techniques were used to characterize the mechanism of BLG aggregation by SY dye. The turbidity and RLS data suggest that BLG form aggregates above 0.07 mM of SY dye concentrations, and aggregate size depends on SY concentrations. Smaller sizes of aggregates formed at lower concentrations of SY, but bigger sizes of aggregates are formed at concentrations above 0.7 mM of SY. Interestingly, the size of aggregates was constant between 0.7–5.0 mM of SY. A similar type of turbidity and RLS profile was found when BLG was treated with tartrazine dye at pH 2.0 [37]. Turbidity results also suggest that the aggregation of BLG is also depends on the pHs of the solutions. Kinetics data suggest that the BLG formed aggregates quickly without the lag phase. The monomer of BLG transformed directly to bigger-sized aggregates within a few seconds. The turbidity, RLS, and kinetics data suggest that BLG aggregations are dependent on SY concentrations. At very low concentrations of SY, aggregation was not seen, but in the presence of higher concentrations, the BLG aggregation was found. The pattern of aggregation was almost the same at all the SY concentrations, but the main difference recorded was that the light scattering is found lower at low concentrations of SY, and the time of saturation was almost the same at every SY concentration. Generally, aggregation kinetics exhibited the characteristic of sigmoidal curves, including the initial lag phase, growth phase, and equilibrium phase. If the aggregation pathways followed sigmoidal curves, it meant that protein aggregation is a fallowing nucleus-dependent phenomenon [38]. Sometimes the length of lag phase, i.e., extrinsic (sequence variants such as mutations, truncation, and extensions) and intrinsic (peptides and proteins, membranes, nanoparticles, and other surfaces, poly-electrolytes, and other polymers, salt, small molecules, pH, temperature), are known to affect the length of the lag phase [39,40]. Some molecules are known to induce protein aggregation without the lag phase and claimed that the monomer was directly converted into aggregates [41]. In our case, the SY induced aggregation escaping the lag phase, the monomer of BLG directly converted into bigger size aggregates instantaneously. From the aggregation kinetics, it was established that some concentrations of SY induce aggregation in BLG quickly. The role of BLG concentrations was also determined in the SY dye-induced aggregation. BLG concentrations also play an important role in the dye-induced aggregation pathways. Further, we have seen the effect of salts on BLG aggregation. The effect of salts on protein aggregation and solubility was much explored [42]. The salts interacted with proteins in three possible ways: (i) non-specific interaction, (ii) specific interactions, and (iii) alteration of water structures by ions. In the non-specific mode of interactions, the salts can reduce the repulsive electrostatic interaction among the protein molecules, decreasing solubility and favoring aggregation. In the specific mode of interaction, the ions of salts interacted with protein and cause salting in or salting-out [43]. In our case, the possible mode of salt interaction is a specific type. The salts and SY are competing for the same site of BLG, so the charges of BLG were not neutralized, and maintained BLG-solvent interaction resultant BLG is soluble. Far-UV CD spectroscopy was utilized to characterize the secondary structure modification in proteins [44]. Plenty of reports are available regarding secondary structure modification during the protein aggregation process [45,46]. Generally, amyloid type aggregates have a β-sheeted, cross β-sheet secondary structure [47]. However, amorphous aggregates do not have a well-defined secondary structure [48]. In our case, we also did not notice any specific transition in the secondary structure except loss in CD signal without a defined peak. The BLG treated with aggregating concentrations of SY dye was reduced and started gaining in the presence NaCl. The negative ellipticity of aggregated samples was completely regained in the presence of salts above 200 mM concentrations, and spectra overlapped the spectra of native BLG. We explored further to detect the morphology of SY dye-induced aggregates, and we found that BLG formed an amorphous type of aggregates.

**Figure 9:**
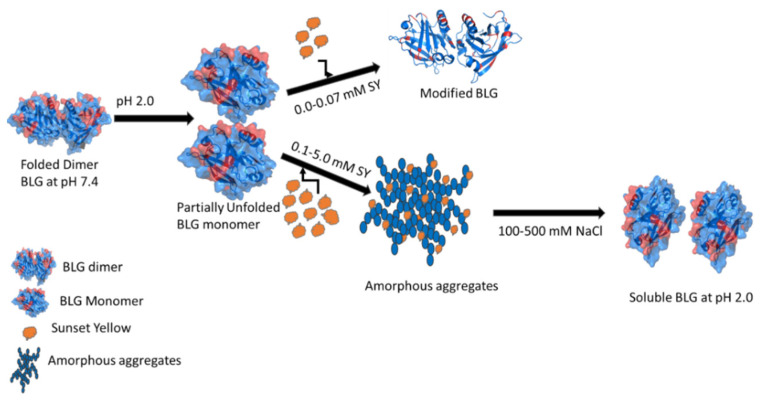
Detail of the mechanism of SY dye-induced BLG aggregation, a schematic presentation.

Figure 9 shows a possible mechanism of interaction of SY to BLG and induces aggregation at acidic pH. The BLG exists in a dimer form at physiological pH because of hydrophobic interaction, and these dimer subunits were dissociated into monomer at pH 2.0 due to electrostatic repulsion [49]. Both the BLG subunits cationic amino acids are protonated, having an overall positive charge (+20) on the surface, and negatively charged residues become neutral at acidic pH. A very low concentration (0.0–0.07 mM) of negatively charged SY was unable to induce aggregation in BLG but slightly modified the overall structure of BLG. However, in the presence of (0.1–5.0 mM) of SY the BLG become aggregated. The sulfate group of SY interacted electrostatically with protonated positively charged amino acids and neutralized the charges on BLG, which leads to aggregation. Due to charge neutralization, the BLG-solvent interaction was perturbed and hydrophobic interaction between BLG molecules is favorable, which makes a suitable environment for BLG aggregation. The SY-induced BLG aggregates were solubilized by the addition of millimolar concentrations of salts “NaCl”. Salts solubilized the aggregates because both the molecules (salts and SY) have an affinity to BLG protein, and electrostatic competition was taking place between SY and salts. The Cl ions of NaCl bind to the protonated positively charged amino acids and diminish the possible electrostatic interaction of the sulfate group of SY dye, resulting in the BLG molecule being soluble.

## 5. Conclusions

We have investigated the effect of SY dye on BLG at acidic pH. We have found that specific concentrations of SY dye accelerated BLG aggregation in vitro. SY dye-stimulated BLG aggregation is fast, and the process of aggregation was achieved within few seconds. The aggregates formed are of an amorphous type, because no specific structure was seen. The far-UV CD spectra of BLG reduced/lost without the conversion/gain into any peak, indicating the amorphous nature of BLG aggregates. The SY-induced BLG aggregation occurred due to electrostatic and hydrophobic interaction. Overall, the results presented here demonstrated that BLG formed amorphous type aggregates when BLG was treated with concentrations above 0.07 mM of SY dye at pH 2.0. Interestingly, salt (NaCl) suppressed the SY-induced aggregates. Electrostatic interactions play a critical role in the suppression of ligand-induced aggregation. This study helps in the understanding of the mechanism of ligand-induced aggregation and suppression.

## Figures and Tables

**Table 1:** Spectroscopic (Turbidity and RLS) data at different experimental conditions.

S. No.	Conditions	Turbidity at 650 nm	Light Scattering at 650 nm
1	BLG (0.2 mg/mL) + 0.0 SY	0.0014 ± 0.0005	0.573 ± 0.094
2	BLG (0.2 mg/mL) + 0.01 mM SY	0.0023 ± 0.0002	0.589 ± 0.096
3	BLG (0.2 mg/mL) + 0.02 mM SY	0.0044 ± 0.0005	0.513 ± 0.016
4	BLG (0.2 mg/mL) + 0.04 mM SY	0.0219 ± 0.001	0.598 ± 1.456
5	BLG (0.2 mg/mL) + 0.05 mM SY	0.0323 ± 0.007	0.631 ± 4.945
6	BLG (0.2 mg/mL) + 0.07 mM SY	0.0779 ± 0.006	0.678 ± 3.478
7	BLG (0.2 mg/mL) + 0.1 mM SY	0.1454 ± 0.028	69.83 ± 5.820
8	BLG (0.2 mg/mL) + 0.5 mM SY	0.4065 ± 0.021	168.91 ± 4.323
9	BLG (0.2 mg/mL) + 1.00 mM SY	0.4179 ± 0.028	165.98 ± 5.455
10	BLG (0.2 mg/mL) + 2.00 mM SY	0.4219 ± 0.018	170.6 ± 7.104
11	BLG (0.2 mg/mL) + 5.00 mM SY	0.4138 ± 0.036	168.63 ± 8.027
12	1.0 mM SY	0.0061 ± 0.0006	0.678 ± 0.034
13	5.0 mM SY	0.0094 ± 0.0008	0.798 ± 0.086

**Table 2:** Percent of α-helix and β-sheet content of βLG were calculated by the K2D2 software at different conditions.

S. No.	Conditions	% α-Helix	% β-Sheet
1	BLG + 0.0 mM SY	13.82	34.61
2	BLG + 0.1 mM SY	4.01	39.08
3	BLG + 0.5 mM SY	3.25	40.55
4	BLG + 1.0 mM SY	4.44	41.17
5	BLG + 2.0 mM SY	3.41	42.08
